# Characterizing alternative and emerging tobacco product transition of use behavior on Twitter

**DOI:** 10.1186/s13104-021-05719-0

**Published:** 2021-08-09

**Authors:** Cortni Bardier, Joshua S. Yang, Jiawei Li, Tim K. Mackey

**Affiliations:** 1 Global Health Policy and Data Institute, San Diego, CA USA; 2 Department of Anthropology, Global Health Program, UC San Diego, 9500 Gilman Drive, La Jolla, CA 92093 USA; 3 S-3 Research LLC, San Diego, CA USA; 4 Department of Public Health, California State University, Fullerton, Fullerton, CA USA

**Keywords:** Tobacco behavior, Electronic cigarettes, Social media, Twitter, Qualitative research

## Abstract

**Objective:**

The objective of this study was to develop an inductive coding approach specific to characterizing user-generated social media conversations about transition of use of different tobacco and alternative and emerging tobacco products (ATPs).

**Results:**

A total of 40,206 tweets were collected from the Twitter public API stream that were geocoded from 2018 to 2019. Using data mining approaches, these tweets were then filtered for keywords associated with tobacco and ATP use behavior. This resulted in a subset of 5718 tweets, with 657 manually annotated and identified as associated with user-generated conversations about tobacco and ATP use behavior. The 657 tweets were coded into 9 parent codes: inquiry, interaction, observation, opinion, promote, reply, share knowledge, use characteristics, and transition of use behavior. The highest number of observations occurred under transition of use (43.38%, n = 285), followed by current use (39.27%, n = 258), opinions about use (0.07%, n = 46), and product promotion (0.06%, n = 37). Other codes had less than ten tweets that discussed these themes. Results provide early insights into how social media users discuss topics related to transition of use and their experiences with different and emerging tobacco product use behavior.

## Introduction

Social media is now a common source of health-related information [1]. This includes user-generated conversations about a variety of topics, with an emerging field focused on better understanding tobacco and alternative and emerging tobacco (ATP) and electronic nicotine delivery system (ENDS) related knowledge, attitudes, and behaviors [2, 3]. User generated social media conversations can be assessed [4] to better understand how health behaviors are changing closer to real-time [5]. This approach introduces certain advantages over traditional survey methodology including faster identification of emerging trends [6]. However, methods to appropriately code social media content for specific health-related topics remain underdeveloped, particularly in the context of characterizing transitions in behaviors that change over time.

Twitter is a micro blogging social networking platform that allows users to tweet 280-character messages, which can then be retweeted, favorited, and shared across a network of online users [7]. Users can form online communities [8] by interacting with other users who share similar beliefs, interests, and opinions about topics. This includes users who initiate, use, and transition between different tobacco and ATP and ENDS products [9, 10]. In fact, Twitter has specifically become a platform for sharing information about electronic cigarettes (e-cigarettes) [11–14] a nicotine delivery device commercially available only in the past decade [15].

Evidencing growing popularity of vaping behavior, studies have shown that online searches for electronic cigarettes have increased [16]. However, increased uptake of different types of e-cigarettes (e.g., Juul, heat-not-burn, etc.), particularly among youth and young adults, has not been without controversy [17]. Ongoing concerns about the long-term health impact of nicotine consumption [18], e-cigarette-related adverse events [19] (e.g., the 2019 outbreak of e-cigarette or vaping product use-associated lung injury), [20–22] and mixed evidence about the efficacy of ATPs as cessation devices, continues to generate public health and patient safety concerns [23, 24]. These concerns are accentuated when trying to assess the interaction of use behavior between traditional combustible tobacco products (e.g., cigarettes, cigars) and ENDS [25].

Understanding the pathways of transition of tobacco and ATP use—including what products users initiate on, why they switch between products, and unique health harms related to dual-use (i.e., simultaneous use of both combustible and ATPs/ENDS)—is still a relatively underdeveloped area of study [26]. Hence, the objective of this study was to examine Twitter user conversations to characterize users’ conversations in relation to transition of use associated with ENDS, with a focus on developing an inductive coding approach specific to characterizing transition of use knowledge, attitudes, and behaviors.

## Main text

### Methods

We conducted a retrospective observational social media study in two phases: (1) data collection; and (2) content analysis using an unsupervised machine learning and inductive coding approach. Inductive content analysis was used to identify and characterize posts relevant to tobacco and ATP use (i.e., “signal” tweets) and involved manual annotation by coders with training in tobacco and substance use behavior, with results used to generate a codebook of transition and behavioral-related themes that could also be iterated on in future social media studies.

### Data collection

Data was first collected from the Twitter public streaming API with a filter to collect all tweets that contained geocoded posts located in the United States, with no further language or demographical restrictions. The time period of data collection was from 07/21/2018–07/21/2019. This initial dataset of geocoded tweets was then filtered for the keywords and hashtags “vape” and “vaping” in order to better isolate relevant twitter posts associated with study aims and for purposes of preliminary data analysis about ENDS behavior. The collected data included textual content of the tweet, user and account information, URLs, and time and date of post.

### Data mining

To identify themes in our full corpus of tweets, we used an unsupervised machine learning approach called the Biterm Topic Model (BTM) designed to detect patterns in data and summarize the entire corpus of tweets into distinct highly correlated categories [27]. BTM is used to sort short text into highly prevalent themes without the need for predetermined coding or training and has been previously used for exploration of key public health topics [28–31]. For each topic, BTM generates the top 20 words that represent the topic cluster. These topics were then reviewed and selected to identify clusters of Twitter conversations relevant to vaping and transition of use. Using BTM, we are able to identify “signal” topics based on the BTM output and eliminate irrelevant topics. BTM topics were first generated after applying keyword filters and were included for further analysis if they were pertinent to vape and vaping behavior, topics were excluded if they contained irrelevant topics or appeared to correlate with non-user generated conversations (e.g., news tweets, etc.)

We then extracted all the posts from the select vaping BTM topics and manually coded the content of tweets in these topics to ensure relevance to user-generated tobacco and ENDS use behavior. Posts were excluded as signals if they were: (1) news related and not organically user-generated content; (2) not written in English; and (3) retweets, the tweets that were retweeted counted as only one tweet. However, all tweets, replies, and tweets containing photos or videos were included to assess additional contextual information in addition to content analysis of text of tweet. Transition of use was classified as switching from one tobacco or ATP/ENDS product to another.

### Content analysis

**Fig. 1:**
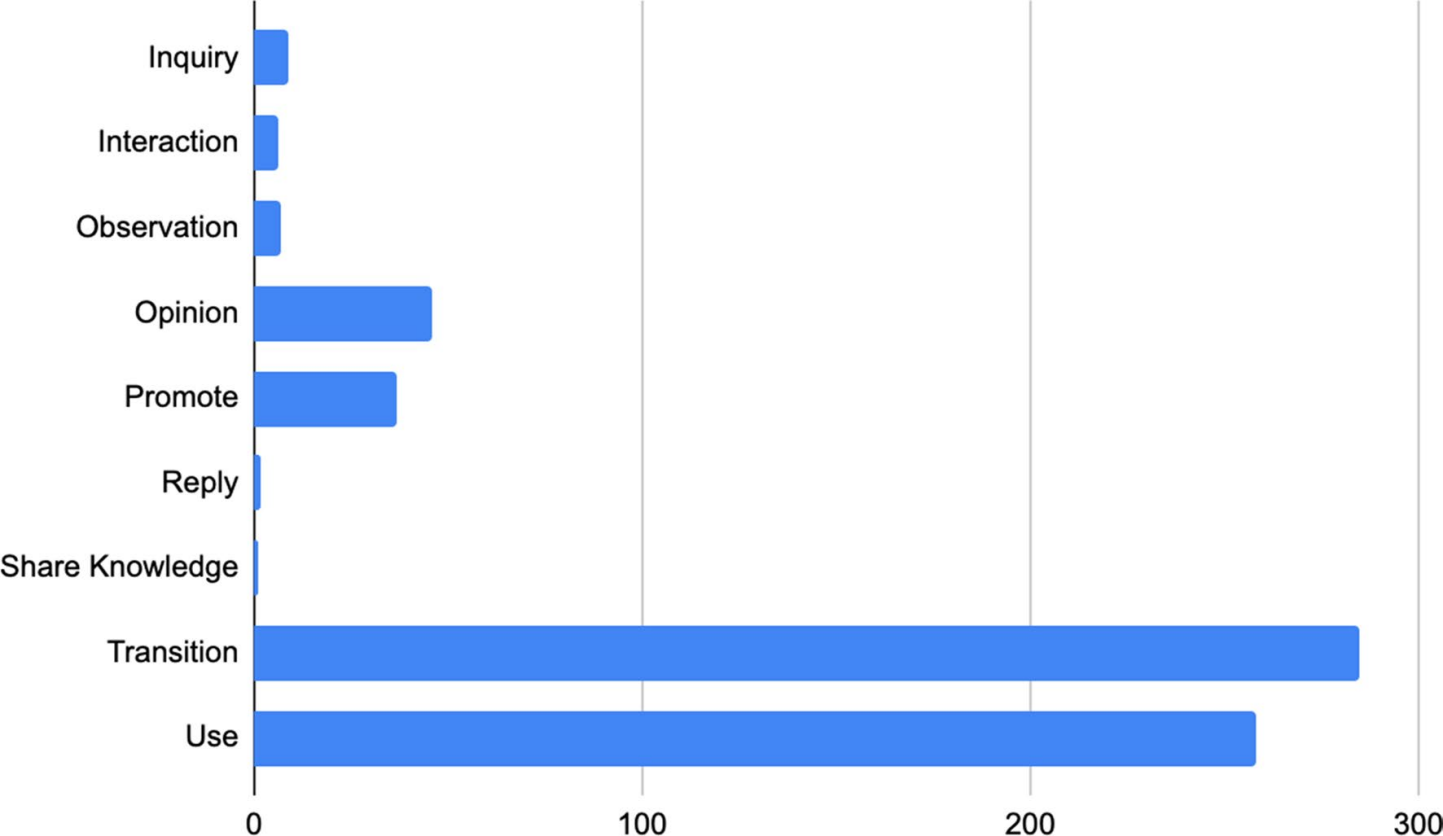
Tweets per tobacco and ATP theme

Tweets and any associated URLs/hyperlinks were aggregated into a table and imported into Atlas.ti qualitative software for content analysis [32]. A first iterative, inductive analysis of the data was conducted (JSY) to identify thematic areas and classify tweets into codes with code descriptions. Tweets were read for identification of thematic areas in the dataset, then coded based on thematic areas of interest. Codes and coding descriptions were developed and modified iteratively throughout the coding process. A second analysis of the dataset was undertaken to expand the codebook to include subcodes. Subcodes and subcode descriptions were created and modified iteratively during a second round of data coding. Once a coding scheme was developed, the data were coded, extracted, and reviewed to assess the validity of the coding scheme by a second coder (CB). The final coding scheme and distribution of codes is presented in Fig. 1 and Table 1.

**Table 1:** Emergent coding scheme for Twitter data for tobacco and ATP use behavior (examples de-identified by paraphrasing)

Code	Sub-code	Definition	Paraphrased Example Tweet
Inquiry (n = 12)		Tweet posing a question to other Twitter users	Is anyone a former e-cigarette user? If so, how did you quit? What healthy habit did you replace it with? Feel free to DM me Is a gas station vape worth purchasing?
Interaction ((n = 6)		Description of an interaction a Twitter user has with another person related to vaping	The security at the club thought my juuls were chewing gum and when I said it was a vape, he said “well that’s embarrassing” I visited my neighbor the other day to try to convince my neighbor to switch from cigarettes to e-cigs, but realized he was smoking cannabis in his backyard instead. It went from a “howdy neighbor” to us smoking together
Observation	General (n = 1)	Observation a person makes related to vaping	At lunch I heard people talking about the best vape pens and anxiety Person 1: I don’t get anxiety Person 2: You are always inebriated; you won’t experience anxiety
	Others vaping (n = 6)	Observation of another person’s vaping behavior	Over the holidays I learned observed that my father began vaping and that’s pretty much it My grandma is learning how to vape because she has medical marijuana now, and I am trying my best not to laugh
Opinion of product	Negative (n = 3)	An opinion of a vaping-related product with negative sentiment	Wax vape = lung hurting juice
	Positive (n = 5)	An opinion of a vaping-related product with positive sentiment	I prefer vapes over juuls please don’t come for me
Opinion	Contradiction (n = 4)	An opinion about vaping the user finds to be a contradiction in some way	@usernameredacted I haven’t been able to refill my vape since cannabis became “legal” it’s ridiculous
	General (n = 6)	General expression of a vaping-related opinion	Yea e-cigs. The addiction is nicotine which is in the vape juice. Humans have been addicted to nicotine, which is actually beneficial. No new findings here
	Humor/sarcasm (n = 2)	An expression of opinion related to vaping meant to be humorous	What grandfather I did not know you vape (redacted url)
	Transition from no use to e-cigarette initiation (n = 7)	Opinion about people who start vaping without doing so as a means to stop smoking	I am highly judging anyone who vapes or juul with nicotine but never began smoking cigarettes. You were never addicted to nicotine. Just trying to be cool
	Vaping as healthier alternative (n = 5)	Opinion about vaping as a healthier alternative to another tobacco-product	Vaping is healthy nicotine
	Vaping helps quit smoking (n = 9)	Opinion about vaping as a method of quitting smoking	Reason number (redacted) why smokers should use vape instead … bonus you won’t smell like a disgusting ashtray upon room entrance. (redacted url)
	Vaping (negative) (n = 6)	General opinion about vaping behavior that has negative sentiment	In my opinion vaping/juuling makes the addiction worse when you’re trying to quit smoking cigarettes
	Vaping as therapeutic (n = 2)	Opinion about the therapeutic benefits of vaping	Do not let anyone dispel your depression because you handle it differently. Each person has a different coping mechanism. If you have depression acknowledge it and seek help (there are great antidepressants) do therapy, vape, cannabis, listen to music. Just own it
	Youth use (n = 6)	Opinion about youth vaping	@redactedusername Yes, I agree. It’s awful children under 18 use e-cigs but it is better than cigarettes. In an ideal world they wouldn’t do neither, but I know when I was a teen I smoked and would have vaped if I could instead
Promotion	Product by user (n = 28)	Promotion of a product as a user of that product	I already have a vape but wow this one is so cool. I’m not biased to the colors or anything
	Product by commercial source (n = 9)	Promotion of a product by a commercial entity	Request your vape! We have the redacted model voice control mod!
Reply (n = 2)		Reply to another Twitter user's inquiry related to vaping	@redactedusername Suorins give a greater headrush and cheaper to refill. 4, 0.7 ml juul pods are the same price as a 30 ml suorin bottle of vape liquid. Lol suorins are an overall better investment
Share nicotine knowledge (n = 1)		Sharing knowledge of e-cigarette nicotine levels	TIL Juul contains 59 mg of nicotine. That’s about 3 × the amount a usual e-cig or vape. They’re not sold in the EU for that exact reason. Wow
Transition	Cannabis to cannabis (n = 3)	Transition between cannabis products	Since I can’t smoke cannabis, I bought a CBD vape and it’s a great substitute
	Cannabis to e-cigarettes (n = 4)	Transition cannabis product to nicotine vaping	I stopped smoking weed but I cannot get rid of my vape. I can’t wait until Tuesday
	Chewing tobacco to e-cigarettes (n = 6)	Transition from chewing tobacco to nicotine vaping	I broke down and bought a vape and flavored liquid. Hopefully this is temporary until I quit chewing tobacco. Wish me luck or tell me I’m dumb for exchanging a bad habit for another (redacted url)
	Cigarettes to e-cigarettes (n = 160)	Transition from conventional cigarettes to nicotine vaping	I feel so much better now that I quit smoking cigs and I hardly use my vape, so I am delighted
	Cigarettes to no product (n = 8)	Transition from conventional cigarettes to no product	Btw I quit smoking cigs as of Tuesday after 10 consistent years. I am doing it cold turkey no nicotine patches or vape pens. So, if I seem tense keep that in mind. I am having surgery next month and nicotine critically reduces your ability to heal
	Cigarettes to vape cannabis (n = 4)	Transition from conventional cigarettes to vaping cannabis product	I take my cbd vape with me everywhere It helped me quit smoking again and keeps the nerves/edge feeling down
	E-cigarette to cannabis (n = 1)	Transition from nicotine vaping to a cannabis product	This is funny but addiction to nicotine isn’t…It’s been 5 weeks and I am so glad I quit. I advise anyone trying to quit to buy cbd vape juice at a vape shop and use it in your mod. It won’t block the withdrawals, but it is similar/relaxing
	E-cigarette to cigarette (n = 7)	Transition from nicotine vaping to conventional cigarettes	What stage of capitalism is that I am using cigarettes since I can’t afford my vape stuff
	E-cigarette to e-cigarette (n = 13)	Transition between nicotine vaping products	I prefer regular vape after about two to three days of salt nic
	E-cigarette to no product (n = 43)	Transition from nicotine vaping to no product	I haven’t used my vape or had caffeine in 2 weeks and I just spent the last 30 min getting yelled at by an elderly tenant without getting a word in. I am so ever the edge right now
	No product to e-cigarettes (n = 17)	Transition from no product to nicotine vaping	You know what’s next? Smoking my vape 24/7 when you’re not a smoker, so awesome dude
	No product to vape cannabis (n = 15)	Transition from no product to vaping cannabis product	According to my husband I am a drug addict now that I got a cbd vape pen
	Unknown product to e-cigarettes (n = 4)	Transition from unknown product to nicotine vaping	Man, I know have slowed down… slowly but surely, I will try a vape soon. I got off of cannabis, I really only smoke black after I drink a brew to get a feeling close to the Mary
Use characteristics	Addiction (n = 21)	Mention of addiction or descriptions of behavior associated with addiction	Attempting to prove that I don’t have an addiction to vape and it’s taking too much strength to not buy a new tip for my tank. I have an addiction,
	Adverse symptoms (n = 25)	Adverse symptom associated with vaping	Hookah and vape are the worst things for me, vaping makes me dry heave
	E-cigarettes complicated to use (n = 1)	Vapes being complicated or hard to use	I almost forgot how to use this 400 vape I bought (redacted url)
	E-cigarettes cost (n = 12)	Cost (either high or low) associated with vaping or cigarettes	Why are the juul starter packs so pricey? For $** I can just buy an actual vape
	Entertainment (n = 3)	Entertaining image or video of vaping	Vape warning! (redacted url)
	Faulty or broken e-cigarette (n = 9)	Vape being faulty or broken or breaking	P.s I stopped smoking cigarettes, and it stresses me that my vape is broken (redacted url)
	Flavor (n = 8)	Flavors used in vape	Flavor of the day is english toffee! delicious
	General (n = 63)	Uncategorized mentions of vaping	I have run out of vape juice and it is horrendous
	Lose or misplace e-cigarette (n = 11)	Misplacing vape, or finding one after having lost or misplaced	I lost my vape pen, damn it lasted so long
	New product (n = 12)	Obtaining or using a new product	I am trying to stop using nicotine and I am now a proud vape owner. I prefer Earl Grey tea flavor. (redacted url)
	Polytobacco/polysubstance (32)	Concurrent (within 30 days) use of two or more forms of a tobacco product or tobacco product and other substance (e.g., alcohol, marijuana)	It’s been a while since I smoked cannabis. I use edibles and vape now Smoking cigarettes at night. Nicotine in vape is not enough
	Reduce use (n = 12)	Reduction in volume of vaping or nicotine used in vaping	No nicotine in my vape I didn’t predict how much my system would be in shock after reducing nicotine use. I moved from vape to juul a couple of days ago and it is very unpleasant
	Stigma (n = 11)	Negative image or reputation associated with vaping	You can make fun of me because I bought a vape
	Therapeutic effects (n = 36)	Therapeutic effects of vaping	My anxiety has been rough lately ***** bought me a CBD vape and it works. It is physically and mentally calming
	Trick (n = 2)	Vaping trick, such as blowing clouds	I have been trying to nail this trick for a while and I finally succeed, I was so shocked. #vape#vapetricks

### Ethics and data collection

Data was collected from the Twitter public API stream and included publicly available tweets that were filtered for posts with geolocation/geotagged information. As the study did not involve human subjects, involved no interactions with online users, and only used publicly available data that was further de-identified for research purposes, ethics, and IRB approval was not required and twitter users were not consented into this study [33]. Any user identifiable information was removed from the study results.

### Results

A total of 40,206 tweets were collected after filtering for “vape” and “vaping” keywords/hashtags. After data filtering, we ran BTM on the keyword filtered data to generate topic clusters and reviewed them for relevance to study aims. We chose 16 BTM clusters, which comprised a total of 5728 (14.25%) tweets selected based on word groupings relevant to vaping and ATP/ENDS behavior terms. After manually annotating these tweets for characteristics relevant to tobacco and ATP/ENDS use and behavior, we removed all non-signal tweets, leaving 589 signal tweets related to transition of use that were further analyzed. The 589 signal posts were categorized into 10 tobacco/ATP/ENDS general use and behavior thematic codes listed and identified in Table 1.

Specific to codes related to transition of use (48.39%, n = 285), thirteen distinct tobacco/ATP/ENDS transition pathways were identified; the term “vaping” was used to describe both nicotine vaping and vaping of cannabis-based products. Transitions detected were cannabis to cannabis (0.005%, n = 3), cannabis to e-cigarettes (0.006%, n = 4), chewing tobacco to e-cigarettes (0.01%, n = 6), cigarettes to e-cigarettes (27.16%, n = 160), cigarettes to no product (0.14%, n = 8), cigarettes to vape cannabis (0.007%, n = 4), e-cigarette to cannabis (0.002, n = 1), e-cigarette to cigarette (0.01%, n = 7), e-cigarette to e-cigarette (0.2%, n = 13), e-cigarette to no product (0.07%, n = 43), no product to e-cigarette (0.03%, n = 17), no product to vape cannabis (0.03, n = 15), and unknown product to e-cigarette (0.007%, n = 4).

There were also transitions among different ATP product types as well as cannabis product types, one of which was vaping a cannabis product. Vaping use factors that were observed as influencing transition of use included self-reporting of addiction prompting use, reaction to adverse symptoms, cost of ATPs/ENDS, faulty or broken ATPs/ENDS, preference for flavors, losing or misplacing ATPs/ENDS, interest in polysubstance use, concern about reducing nicotine levels, stigma, and the alleged therapeutic effects of vaping, especially cannabis.

## Discussion

This study explored user-generated conversations occurring on Twitter in relation to tobacco and ATP/ENDS use, with a specific focus on transition of use between these highly addictive products. We observed that this subset of Twitter users actively tweeted about their experience using tobacco and ATPs/ENDS, representing powerful information about this behavior that is influenced by a changing landscape of new and emerging nicotine products. The majority of tweets reviewed related to tobacco and ATP/ENDS use and behavior characteristics, including users asking about tobacco/ATP/ENDS products, how to quit, observations of tobacco/ATP/ENDS use behavior, opinions about products and vaping (including claiming vaping as a healthier alternative to tobacco or its alleged therapeutic benefits), sharing knowledge about tobacco/ATP/ENDS products, and specific characteristics of use (e.g. addiction, adverse events, costs, flavoring, tricks, etc.)

Close to half of all conversations discussed transition of use behavior, including users actively discussed the types of tobacco/ATP/ENDS products used and switched between, as well as provided reasons for product use change. A wide variety of tobacco/ATP/ENDS products were mentioned, including combustible tobacco products (e.g., cigarettes), chewing tobacco, different types of e-cigarettes (Juul, vaping pens, etc.) and cannabis smoking products. Transition was observed between different products and within specific product classes (i.e., transitioning from one type of e-cigarette product to another), with some users (n = 32) self-reporting polytobacco and polysubstance behavior (e.g., smoking cigarettes and also vaping). Users expressed various sentiment about different products including how products could act as substitutes for others, what products made them feel better, attempts to quit use of one product by switching to another, and issues related to cost and access. Some users stated that cannabis vaping products helped them with cessation of nicotine addiction.

Based on these preliminary results, Twitter appears to enable robust conversation and sharing of information related to tobacco and ATP/ENDS use and can act as a digital forum for smokers and vapers to accumulate knowledge, share experiences, and actually lead to potential behavior change associated with nicotine use and addiction.

## Conclusion

The results of our study are exploratory in nature and were derived from a sample of general geolocated tweets over a one-year period, which were then filtered for common vaping keywords and then analyzed using unsupervised machine learning. The results of this study are not generalizable to overall trends in tobacco or ATP/ENDS behavior, but nevertheless provide important insights into conversations occurring among Twitter users specific to transition of tobacco and nicotine product use. Themes associated with the transition of use were primarily focused on navigating quit attempts or having trouble quitting in the past, those who had relapsed to nicotine addiction, and those who had quit cigarettes but still vaped. These results provide early evidence that experiences in transition of use also present opportunities for more targeted cessation interventions, particularly in the context of increasing knowledge of known health harms related to tobacco use and nicotine addiction and exposure [34, 35]. Future work should conduct further confirmatory studies to assess if themes related to transition of use knowledge, attitudes and behaviors observed hold true in other digital communities and use more structured research approaches to generalize findings. Future studies should also examine other platforms now popular among youth and young adults, such as Instagram, Snapchat, and TikTok.

## Limitations

This study was exploratory and meant to generate hypotheses for future research. The study’s limitations include use of a single platform and that Twitter user demographics may not reflect that of the general population of tobacco/ATP/ENDS users. The sample of tweets were also limited based on a convenience sample generated from geocoded tweets, and hence, may be subject to sample bias as it is estimated that only 1% of all tweets are geocoded [36, 37]. Future studies should use multiple Twitter APIs to generate a more representative Twitter dataset.

## Data Availability

The de-identified data that support the findings of this study are available upon request to corresponding author tmackey@ucsd.edu and certain data will be available freely from the website www.ghpolicy.org.
